# Author Correction: Acupuncture elicits neuroprotective effect by inhibiting NAPDH oxidase-mediated reactive oxygen species production in cerebral ischaemia

**DOI:** 10.1038/s41598-021-98780-5

**Published:** 2021-09-27

**Authors:** Guang-Xia Shi, Xue-Rui Wang, Chao-Qun Yan, Tian He, Jing-Wen Yang, Xiang-Hong Zeng, Qian Xu, Wen Zhu, Si-Qi Du, Cun-Zhi Liu

**Affiliations:** Acupuncture and Moxibustion Department, Beijing Hospital of Traditional Chinese Medicine Affiliated To Capital Medical University, 23 Meishuguanhou Street, Dongcheng District, Beijing, 100010 China

Correction to: *Scientific Reports* 10.1038/srep17981, published online 10 December 2015

This Article contains errors. Data presented in Figure 1A was previously reported by the authors in Figure 1A of Reference^1^, which is not cited in the Article.
